# Substrate-Induced Changes on the Optical Properties of Single-Layer WS_2_

**DOI:** 10.3390/ma16072591

**Published:** 2023-03-24

**Authors:** F. D. V. Araujo, F. W. N. Silva, T. Zhang, C. Zhou, Zhong Lin, Nestor Perea-Lopez, Samuel F. Rodrigues, Mauricio Terrones, Antônio Gomes Souza Filho, R. S. Alencar, Bartolomeu C. Viana

**Affiliations:** 1 Instituto Federal de Educação, Ciência e Tecnologia do Piauí-Campus Campo Maior, Avenida Raimundo Doca da Silva, S/N-Fazendinha, Campo Maior 64280-000, Piauí, Brazil; 2 LIMAV—Laboratório Interdisciplinar de Materiais Avançados, Programa de Pós-Graduação em Engenharia e Ciência dos Materiais (PPGCM), Universidade Federal do Piauí, Teresina 64049-550, Piauí, Brazil; 3 Instituto Federal de Educação, Ciência e Tecnologia do Maranhão-Campus Alcântara, Alcântara 65250-000, Maranhão, Brazil; 4 Programa de Pós-Graduação em Engenharia de Materiais (PPGEM), Instituto Federal de Educação, Ciência e Tecnologia do Maranhão-Campus Monte Castelo, Avenida Getúlio Vargas, Nº 04, São Luís 65030-005, Maranhão, Brazil; 5 Department of Physics and Center for 2-Dimensional and Layered Materials, The Pennsylvania State University, University Park, PA 16802, USA; 6 Departamento de Física, Universidade Federal do Ceará, Fortaleza 60455-900, Ceará, Brazil; 7 Faculdade de Física, Universidade Federal do Pará, Belém 66075-110, Pará, Brazil; 8 Departamento de Física, Campus Ministro Petrônio Portella, Universidade Federal do Piauí, Teresina 64049-550, Piauí, Brazil

**Keywords:** tungsten disulfide, 2D materials, optical properties

## Abstract

Among the most studied semiconducting transition metal dichalcogenides (TMDCs), WS${}_{2}$ showed several advantages in comparison to their counterparts, such as a higher quantum yield, which is an important feature for quantum emission and lasing purposes. We studied transferred monolayers of WS${}_{2}$ on a drilled Si${}_{3}$N${}_{4}$ substrate in order to have insights about on how such heterostructure behaves from the Raman and photoluminescence (PL) measurements point of view. Our experimental findings showed that the Si${}_{3}$N${}_{4}$ substrate influences the optical properties of single-layer WS${}_{2}$. Beyond that, seeking to shed light on the causes of the PL quenching observed experimentally, we developed density functional theory (DFT) based calculations to study the thermodynamic stability of the heterojunction through quantum molecular dynamics (QMD) simulations as well as the electronic alignment of the energy levels in both materials. Our analysis showed that along with strain, a charge transfer mechanism plays an important role for the PL decrease.

## 1. Introduction

Transition metal dichalcogenides have received considerable attention of scientific community from several fields since the beginning of 2010s [1]. However, among more than 40 transition metal dichalcogenides (TMDCs) presenting a plethora of physical and chemistry properties [2], four of them (MoS${}_{2}$, WS${}_{2}$, MoSe${}_{2}$ and WSe${}_{2}$) have received most of the efforts due to their semiconducting properties, ambient stability and its indirect to direct band gap transition in the monolayer limit [3]. In spite of only MoS${}_{2}$ and WS${}_{2}$ being found in nature, many applications using TMDCs were reached so far, such as solar cells [4], field-effect transistors (FETs) [5], and photodetectors [6]. It is known that monolayer WS${}_{2}$ shows certain advantages compared with other TMDCs, owing its larger spin orbit coupling [7], which makes WS${}_{2}$ attractive for spintronics applications, due to the strong photon emission [8] along with a flexible doping character, being n-type [9] or p-type depending on its growth conditions [10], endowing it with potential application in optoelectronics. All these findings along with the fact that W is a commodity more abundant than Mo and less toxic as well [11], these features make 2D WS${}_{2}$ a promising candidate for further fundamental research and technological applications.

Monolayers of W-based TMDCs have attracted attention of researchers due to their outstanding photonic properties. Wu et al. [12] transferred monolayer WSe${}_{2}$ onto a prefabricated gallium phosphide substrate where nanocavities were drilled. They observed a sharp photoluminescence peak at 739.7 nm, thus indicating that a lasing behavior might be obtained from TMDC monolayers associated with quantum confinement. Ye et al. [13] studied WS${}_{2}$ onto drilled Si${}_{3}$N${}_{4}$ substrate and demonstrated that a spike in the photoluminescence spectrum is observed at 612.2 nm. They also reported that WS${}_{2}$ presents a higher quantum yield relative to other TMDCs, thus indicating another advantage for using WS${}_{2}$. Differing from above mentioned studies, where the measurements where based on low temperatures, Shang et al. [14] managed to measure a highly concentrated emission nearby 639.5 nm at room temperature, reinforcing the importance of these materials to optoelectronics applications. However, an explanation for Si${}_{3}$N${}_{4}$ substrate and WS${}_{2}$ interaction based on elementary electronics and semiconductor physics definitions still lacks.

According to Kim and coworkers [15], that also verified quantum emission in WSe${}_{2}$ recently using a different approach without drilled substrates, the quantum emission properties are far from a complete understanding. In this sense, we study experimentally the WS${}_{2}$ monolayer transferred onto drilled Si${}_{3}$N${}_{4}$ substrate, in order to have insights about the influence of the substrate over the WS${}_{2}$ optical properties through Raman and photoluminescence measurements. Our experimental results clearly show a considerable quenching of the photoluminescence (PL) emission for supported WS${}_{2}$ in comparison to the suspended (over the hole) one. In addition, we carried out DFT-based calculations aiming to better understand the electronic properties of the Si${}_{3}$N${}_{4}$–WS${}_{2}$ semiconductor heterojunction. First, we performed molecular dynamics (MD) calculation in order to verify the feasibility of the junction face to the significant strain that the Si${}_{3}$N${}_{4}$ substrate might impose to WS${}_{2}$ lattice, further confirming the strong in-plane bonding on tungsten-based TMDCs [15]. Furthermore, we also developed an analysis of the band alignment prior to the junction according to the semiconductor theory, which was confirmed by electronic band structure calculations via density functional theory (DFT). Our model points out that the differences in PL emission, in this case, are associated with charge transfer due to orbital overlapping resulting from strain, thus creating highly confined regions of the active semiconductor.

## 2. Materials and Methods

### 2.1. Synthesis of WS_2_ Single-Layer

The single-layer WS${}_{2}$ was synthesized by a halide-assisted atmospheric pressure APCVD method in order to facilitate the transport of the tungsten source toward the substrate used for the crystal growth as described in Ref. [16]. A mixture of 8 mg of WO${}_{3}$ powder and 2 mg of NaBr powder were loaded on the bottom of an aluminium crucible and a large piece of freshly cleaned SiO${}_{2}$/Si was then placed on the top of the crucible to serve as the growth substrate. The crucible was placed into the center of the furnace, and another melting pot containing 400 mg of sulphur powder was placed on the upstream. During the synthesis, the furnace temperature was increased up to 825 ${}^{{}^{\circ}}$C during 30 min, and maintained at that temperature for 15 min (again, in the meantime, the sulphur was heated up separately to 250 °C by a heating belt). During the whole process, 200 sccm of argon was used as the carrier gas.

### 2.2. Transference of Single Layer WS_2_ to the Drilled Substrates

**Figure 1:**
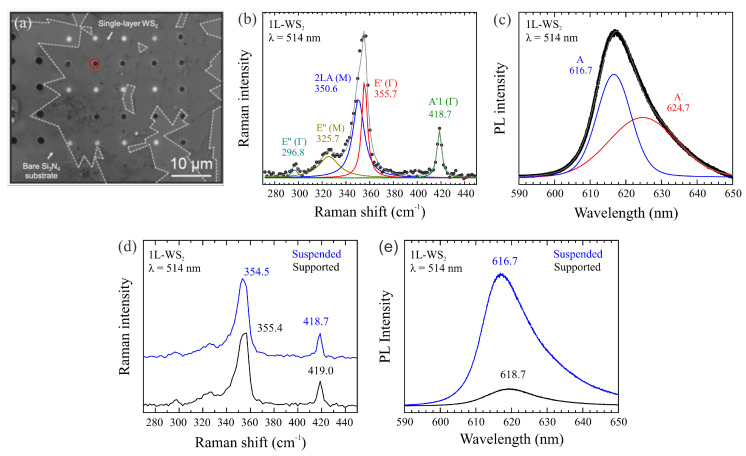
(**a**) WS${}_{2}$ sample transferred over the drilled Si${}_{3}$N${}_{4}$ substrate. (**b**) Raman spectrum for 1L-WS${}_{2}$ as synthesized on SiO${}_{2}$/Si substrate excited with 514 nm laser line. (**c**) PL spectrum of 1L-WS${}_{2}$ as synthesized on SiO${}_{2}$/Si substrate. (**d**) Comparison of Raman spectra of supported (blue trace) and suspended (black trace) regions of 1L-WS${}_{2}$ transferred. The huge discrepancy in PL intensity for supported and suspended 1L-WS${}_{2}$ regions is shown in (**e**). The red circle was the region were the measurements were acquired.

We have used as substrate for transference an Si chip with Si${}_{3}$N${}_{4}$ membrane window. The Si${}_{3}$N${}_{4}$ substrate has holes of 5–10 μm in diameter drilled by means of focused ion beam (FIB) attached to a scanning electron microscope (SEM). A thin layer of poly(methyl methacrylate) (PMMA) (495K, A4) was first spin-coated for 1 min onto the as-grown WS${}_{2}$ samples, with a speed of 2000 rpm. After curing, the set substrate/as-growth WS${}_{2}$/PMMA was etched in a 2 M NaOH solution for about 30 min to lift off the PMMA layer that was coating the samples. Afterward, the film detached during the process was rinsed in distilled water for several times and fished onto the drilled substrates. The substrates were then cleaned in acetone and isopropyl alcohol (IPA) to remove PMMA residues. Figure 1a shows an optical image of WS${}_{2}$ single-layers over the drilled substrate.

### 2.3. Raman and Photoluminescence Experiments

Raman spectra were acquired by using a Renishaw inVia confocal microscope-based spectrometer with 514 nm laser excitation in a back scattering geometry. The laser power was adjusted in 20 μW in order to avoid possible heating and damage of the sample, and to improve the signal-to-noise ration in the spectra. A 100× objective lens with numerical aperture (NA) of 0.95 was used to focus the laser beam and to collect the scattered Raman signal. It is also important to emphasise that all PL measurements were also performed in the same set up of Raman experiments.

### 2.4. First Principles Calculations

Our calculations were entirely developed within the DFT formalism [17,18] through its implementation in the Spanish Initiative for Electronic Simulations with Thousands of Atoms (SIESTA) package [19]. Numerical atomic orbitals (NAOs) are implemented in the code [20] in order to represent the outermost orbitals of each atomic species, in this case, a double zeta plus polarization (DZP) basis set was chosen. For the exchange–correlation interaction, we use the Generalized Gradient Approximation (GGA) according to Perdew–Burke–Ernzerhof (PBE) parametrization [21]. The contribution from the electrons in atomic core are taken into account using the norm conserving pseudopotential technique as proposed by Troullier–Martins [22]. The Brillouin zone is sampled though a Monkhorst–Pack-like grid [23] of 5 × 5 × 1 at least for folded cells. The numerical grid for the integrals solutions is also set with a 300 Ry mesh cutoff, while the self-consistent cycle is considered converged when a threshold of 0.1 meV is reached. When applied, the atomic positions are relaxed until the interatomic forces are below 0.01 eV/Å.

The quantum molecular dynamics (QMD) simulations were performed employing the same parameters presented above. The thermostat formalism proposed by Shuichi Nosé [24] is used to control the temperature at 300 K along the run. Every time step is set as 1.0 fs and the simulations are allowed to run over than 3.0 ps. The final structure at the end of MD simulations is the one taken to perform the relaxation to minimize the energy in the DFT calculation.

## 3. Results and Discussion

As mentioned before, Figure 1a shows a wide-view optical image of the 1L-WS${}_{2}$ sample transferred onto the drilled Si${}_{3}$N${}_{4}$ substrate, and edges of 1L-WS${}_{2}$ flakes are marked with white dashed lines. Figure 1b shows Raman spectrum of 1L-WS${}_{2}$, excited with 514 nm wavelength, as synthesized on SiO${}_{2}$/Si substrate before the transference process. It presents five vibration modes with irreducible representation and Raman frequency as follows: E${}^{\prime \prime }$(Γ) (296.8 cm${}^{-1}$), E${}^{\prime \prime }$(M) (325.7 cm${}^{-1}$), 2LA(M) (350.6 cm${}^{-1}$), E${}^{\prime }$(Γ) (355.7 cm${}^{-1}$) and A${}^{\prime }$${}_{1}$(Γ) (418.7 cm${}^{-1}$) [25]. A comparison of our Raman data with those reported in the literature and our results is shown in Table 1. Our findings for WS${}_{2}$ single-layer are in good agreement with previously published data, even when compared to theoretical results, as seen in Ref. [26], where the A${}^{\prime }$${}_{1}$ mode was predicted to be at 418.7 cm${}^{-1}$ thus slightly different from our measurement. Moreover, the enhancement in the intensity of double resonance 2LA(M) mode matches well with the previous work on single-layer WS_2_, further confirming the monolayer nature of our APCVD-synthesized WS_2_ flake [27].

Figure 1c shows the PL spectrum of 1L-WS${}_{2}$ as synthesized on SiO${}_{2}$/Si substrate. It is characterized by a strong peak centered at 616.7 nm, assigned to a neutral exciton (A), and a shoulder centered at 624.7 nm, assigned to a trion (A${}^{-}$) [30,31]. Figure 1d,e compare the 1L-WS${}_{2}$ Raman and PL spectra acquired in the supported and suspended regions (i.e., over the Si${}_{3}$N${}_{4}$ substrate and on the hole, respectively). It is observed that the Raman modes (PL bands) are slightly redshifted (blueshifted) for the suspended sample compared with the supported one, thus indicating a difference of tensile strain between both regions higher than 1% [30,32]. It is worth mentioning a pronounced quenching of PL in the supported regions for 1L-WS${}_{2}$. More details about the measurements may be found in the available Supporting Information.

**Figure 2:**
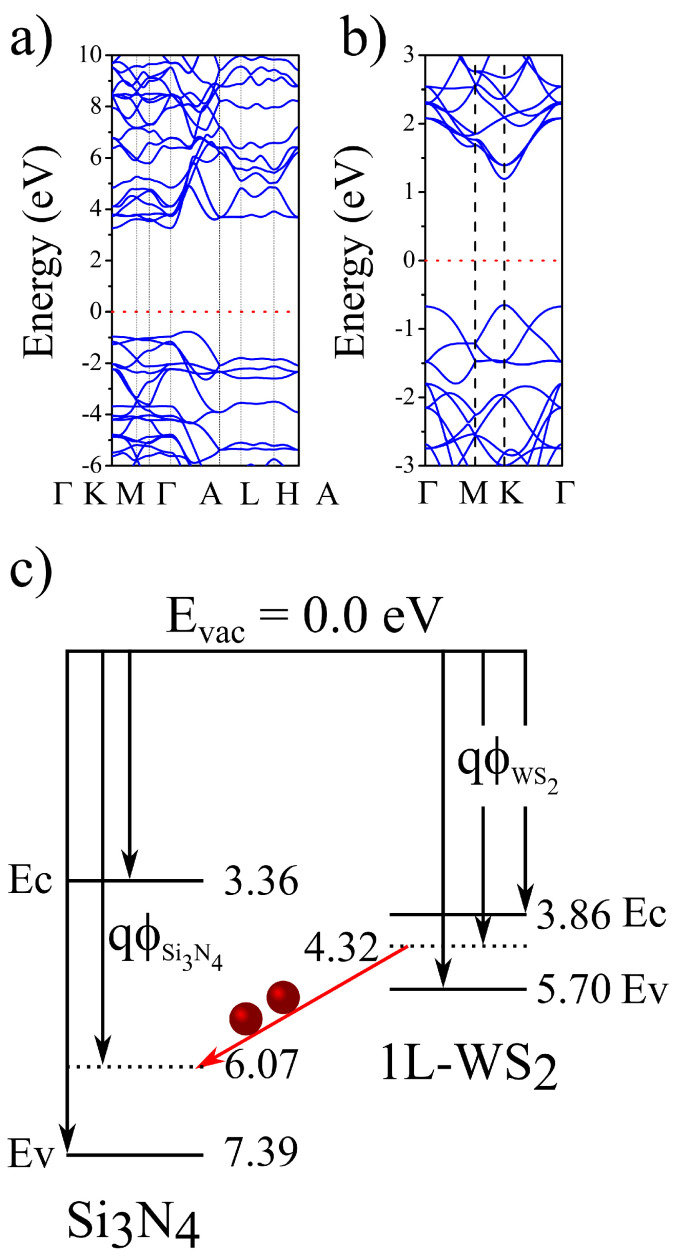
The electronic energy dispersions for bulk Si${}_{3}$N${}_{4}$ and 1L-WS${}_{2}$ are shown in (**a**,**b**), respectively. The Si${}_{3}$N${}_{4}$ substrate is found to be a wide gap insulator with an indirect band gap while the 1L-WS${}_{2}$ is known as a direct band gap semiconductor. The panel in (**c**) shows an electronic band diagram for each material, indicating a trend for a charge transfer from WS${}_{2}$ to the Si${}_{3}$N${}_{4}$ substrate. Each value in (**c**) has been multiplied by −1 for sake of convenience. Here, E${}_{v}$ and E${}_{c}$ stand for the energies of valence and conduction bands in this same order.

In order to understand such a pronounced PL quenching, we perform DFT calculations. First, we analyse separately the Si${}_{3}$N${}_{4}$ and WS${}_{2}$ electronic energy band structure. For Si${}_{3}$N${}_{4}$, Figure 2a, our model has predicted a lattice vector of 7.72 Å, which is in a good agreement with the reported experimental value of 7.75 Å [33,34,35]. The indirect band gap has been evaluated as 4.03 eV, within GGA approximation. The band structure presents its conduction band minimum around the Γ-point. On the other hand, in plane a flat band is observed in the Brillouin zone path Γ→K→M→Γ while the valence band maximum is predicted to be between the Γ and A points, which are also in a good agreement with results reported in Ref. [35]. Furthermore, we also calculate the work function $\Phi_{Si_{3}N_{4}}$ of Si${}_{3}$N${}_{4}$ substrate according to its definition $\Phi =E_{vac}-E_{f}$ at 6.07 eV, here $E_{vac}$ stands for the vacuum level energy and $E_{f}$ for the Fermi level.

The energy dispersion for 1L-WS${}_{2}$ is shown in Figure 2b. As expected, a direct band gap of 1.84 eV at K point is obtained, in good agreement with previous DFT calculations [36]. We also calculate the work function for WS${}_{2}$ and its value ($\Phi_{WS_{2}}$ = 4.32 eV) agrees well with previous data published elsewhere [37]. In Figure 2c, we show the band diagrams for Si${}_{3}$N${}_{4}$ and 1L-WS${}_{2}$ separately. One can observe the 1L-WS${}_{2}$ Fermi level lies above the Si${}_{3}$N${}_{4}$ one. In an idealized situation, after the contact of both semiconductors the electrons have a natural tendency to flow from the monolayer WS${}_{2}$ to the Si${}_{3}$N${}_{4}$ substrate. This trend combined to the indirect band gap shown in Si${}_{3}$N${}_{4}$ might be one of the factors contributing to the PL quenching. However, it is known that the TMDCs are quite sensitive to strain. Since the lattice mismatch between the monolayer and the substrate naturally lead to strain, we further investigate theoretically the WS${}_{2}$/Si${}_{3}$N${}_{4}$ vertical heterojunction.

**Figure 3:**
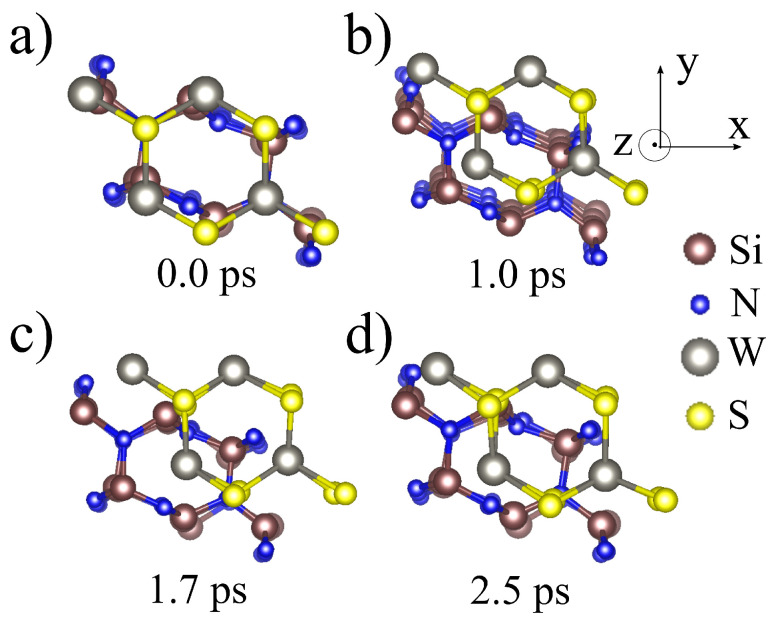
Snapshots from Nosé-Thermostat molecular dynamics of 1L-WS${}_{2}$ over Si${}_{3}$N${}_{4}$ substrate stacked in a AA pattern, as shown is (**a**). Along the MD evolution, the 1L-WS${}_{2}$ slowly deviates from AA stacking towards the AB, as featured in (**b**,**c**). The heterojunctions reach an energy minimum at 2.5 ps, as depicted in (**d**), remaining on this site for the rest of the simulation, allowed to run for over than 3.0 picoseconds.

Aiming to have more insights about the interaction between 1L-WS${}_{2}$ and Si${}_{3}$N${}_{4}$ substrate, we first investigate the thermodynamic stability of that heterojunction. We place the 1L-WS${}_{2}$ on top of a slab of Si${}_{3}$N${}_{4}$ substrate. We found that the slab of five unit cells in the vertical direction is enough to mimic the structural properties of bulk Si${}_{3}$N${}_{4}$. The initial geometry is shown in Figure 3a, where we see that the molecular dynamics simulation was initialized in a stacking sequence that resembles the AA stacking for other 2D materials. The initial configuration at the beginning of the molecular dynamic was chosen according to an E × h curve, (See the Supporting Information (SI) Figure S2) where E stands for the total energy of the system and h for the vertical distance between the outermost plans of the heterojunction. The calculation was allowed to run for more than 3 picoseconds, where each MD step was set in 1 femtosecond. By looking at Figure 3b,c, it is possible to infer that the 1L-WS${}_{2}$ started a transition from the AA stacking to an AB staking where the tungsten atom is close to the center of Si${}_{3}$N${}_{4}$ ring. At 2.5 ps, Figure 3d, the 1L-WS${}_{2}$ has drifted to an energy minima remaining at this positions up to the end of the run around 3.5 ps. Our MD calculations predict that WS${}_{2}$ lattice shall not melt in spite the maximum strain that the substrate may cause, thus indicating that the grow of WS${}_{2}$ monolayer is feasible on top of Si${}_{3}$N${}_{4}$, once more evidencing the strong in-plane bonding arrangement for W-based TMDCs. Furthermore, our simulation indicated that the sulphur atom strongly interacts with the silicon site on the Si${}_{3}$N${}_{4}$ substrate.

**Figure 4:**
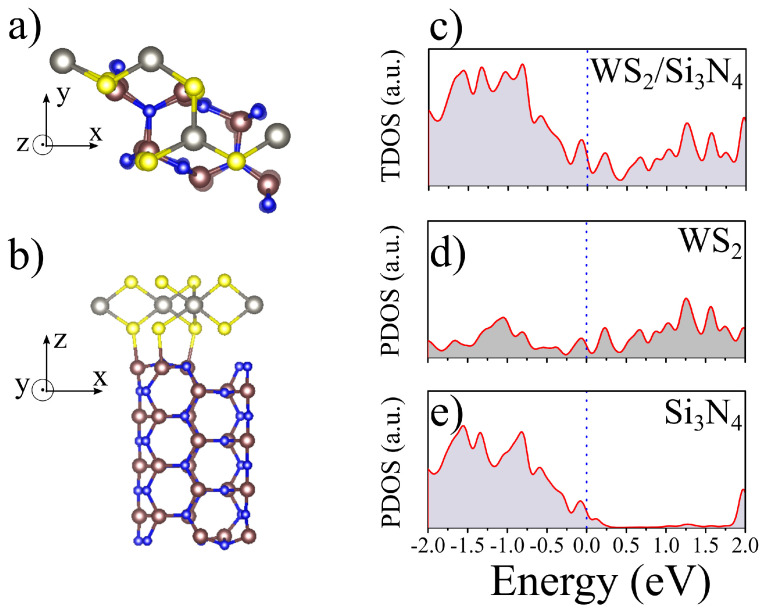
The relaxed atomic positions from the MD run are shown in (**a**) top and (**b**) side views of the heterojunction. The total density of states (TDOS) of the system is depicted in (**c**), while the partial density of states (PDOS) due to the WS${}_{2}$ fraction is shown in (**d**), and the PDOS for Si${}_{3}$N${}_{4}$ substrate is shown in (**e**).

Taking the structure from the last MD step, we relaxed the atomic positions and the lattice vectors of the unit cell, as shown in Figure 4a. The final average interplanar distance between WS${}_{2}$ and Si${}_{3}$N${}_{4}$ substrate is predicted to be 3.03 Å. The lateral view of the unit cell is shown in Figure 4b, where it is possible to see that the S atom from the bottom sublattice of WS${}_{2}$ is strongly interacting with the outmost Si atom from the substrate (the S-Si distance is of 2.42 Å). The vertical distance of the two S atoms in different sublattice of monolayer WS${}_{2}$ is 3.19 Å. However, the Si${}_{3}$N${}_{4}$ substrate exerts considerable distortions over the WS${}_{2}$ monolayer. The S-S distance in our unit cell is calculated to be 2.80 Åwhen the lower S atom is interacting to the Si atom, and to be 2.90 Å when there is no interaction. Such increase in the overlap of pz orbitals of sulphur atoms in few-layers and bulk WS${}_{2}$ is known to be the cause of a direct to indirect transition of the band gap, or even metalization [38,39].

The lattice mismatch strain in the heterojunction has consequences to the electronic structure of the system as seen in the total density of states shown in Figure 4c, which shows an increase in the density of states nearby the Fermi level. Such increment is also featured for high pressures situations [39] that leads to a increase in the carrier concentration next the Fermi level. In other words, the Si${}_{3}$N${}_{4}$/WS${}_{2}$ heterojunction might be a system where features only obtained at high pressures may be reached at ambient pressure by exploiting the lattice mismatch. Further analysis of the partial density of states (PDOS) shows that most carrier concentration increment is occurring on the WS${}_{2}$ side, as seen in Figure 4d. As expected from the band diagram in Figure 2c, the PDOS for Si${}_{3}$N${}_{4}$ presents a shift of the band gap, also exhibiting a slight increment of the density of states around the Fermi level as seen in Figure 4e. All these theoretical findings are key factors to explain the experimental results related to the PL quenching presented in Figure 1e. However, we can look at the charge transfer mechanism in this heterojunction, in order to better understand the PL quenching observed for supported regions.

**Figure 5:**
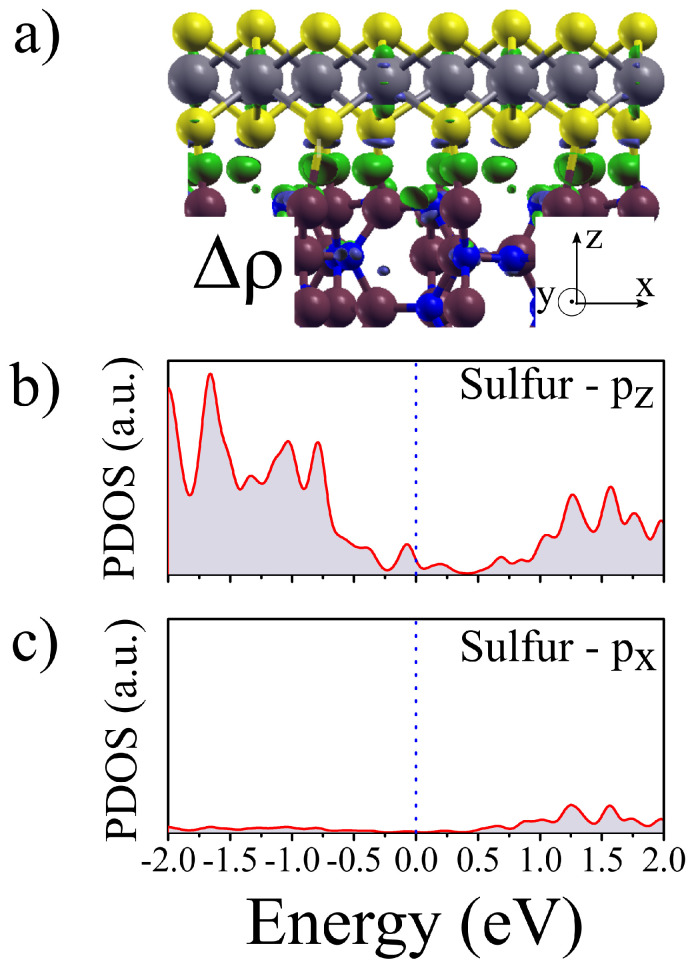
(**a**) Charge difference Δρ exchanged between WS${}_{2}$ and Si${}_{3}$N${}_{4}$ substrate. (**b**) PDOS for the sulphur *pz* orbitals populating around the Fermi level. (**c**) *px* from sulphur showing no reactivity for energies nearby E${}_{F}$, thus indicating that the in plane bonds are still present.

Figure 5a shows the charge difference calculated as $\Delta \rho =\rho_{junction}-\rho_{WS_{2}}-\rho_{Si_{3}N_{4}}$. We emphasize at this point that seeking to mitigate the basis set superposition error (BSSE), Δρ was calculated according to the ghost atom technique [40]. Therefore, it is possible to observe that charge is transferred in the system (green isosurface), not only on sites where the Si-S interaction but also occurs throughout the entire interface. We finally check the partial DOS related to sulphur *p* orbitals. Figure 5b shows the PDOS for the S *pz* component, where one might observe that the increment of DOS nearby the Fermi level is due to *pz* orbital. On the other hand, as expected the in-plane components of sulphur (*px*), specifically shown in Figure 5c, does not present DOS close to the Fermi level, thus evidencing that despite the strain, the in-plane bonds to the transition metal (W) are still preserved. All these factors contribute to the quenching of the PL signal observed in our experiments. Actually, it has been observed that strain only is not enough to quench the PL signal [30]. Thus, substrate–sample interaction is an important parameter for PL quenching as observed in our experiments and validated by the model. Therefore, from all the findings discussed so far, it is possible to state that the strain on the 1L-WS${}_{2}$ led by the Si${}_{3}$N${}_{4}$ substrate results in new orbital overlaps in the dichalcogenides system. Such hybridization along with the natural trend of the electrons to flow from WS${}_{2}$ to Si${}_{3}$N${}_{4}$ substrate, which is a semiconductor of indirect gap makes the considerable decrease in the PL measurements for supported regions.

## 4. Conclusions

In summary, our results showed experimentally that 1L-WS${}_{2}$ interacts with Si${}_{3}$N${}_{4}$ substrate since the Raman spectra (PL spectra) present a redshift (blueshift) of ~1 cm${}^{-1}$ (~2 nm), which evidences an existence of some level of strain in the samples. Besides, a quenching of the photoluminescence intensity for supported regions by the substrate as compared to suspended regions was observed. Calculations based on DFT showed that the energy band alignment between electronic levels from the substrate and the TMDC played an important role in PL quenching. For the case of Si${}_{3}$N${}_{4}$–WS${}_{2}$ heterojunction, the electrons tend to flow from WS${}_{2}$ to Si${}_{3}$N${}_{4}$ substrate for Fermi level equilibrium sake. This charge transfer process between both materials leads to an increased density of states nearby the Fermi level, striking down the exciton formation in these areas. The WS${}_{2}$ spots over a drilled hole turned into confined regions where PL remained. Therefore, not only strain is the cause for a decreased PL in TMDCs on substrate, but also the charge transfer mechanism associated with the alignment of the energy levels in the substrate and TMDC is also a key factor in this process. From all the analysis presented in this study, it is fair to conclude that causes of PL quenching in the TMDCs over Si${}_{3}$N${}_{4}$ substrate are a combination of strain, which led to an overlap of the orbitals in WS${}_{2}$ for this case, and the natural trend of electrons to flow from a direct gap semiconductor into an indirect one. Furthermore, the unstrained regions over the holes turned into confined areas where lasing conditions may be found, as seen in previous publications elsewhere [12,13,14,15].

## Figures and Tables

**Table 1:** Comparison of Raman shift (cm${}^{-1}$) obtained in this work with their counterparts of different types of WS${}_{2}$ samples reported in the literature.

Paper	2LA(M)	A${}^{\prime }$${}_{1}$	Type
This work	350.6	418.7	triangle
Gutiérres et al. [28]	-	417.5	triangle
Peymioo et al. [8]	350.0	417.0	triangle
Zhao et al. [29]	-	418.0	flake
Berkdemir et al. [27]	352	417.5	triangle
Terrones et al. [26]	-	418.7	unit cell ^1^

^1^ DFT.

## Data Availability

Not applicable.

## Supplementary Materials

The following supporting information can be downloaded at: https://www.mdpi.com/article/10.3390/ma16072591/s1, Figure S1: (a,b) Optical and fluorescence microscopy images of a typical CVD-grown WS${}_{2}$ flake. The strong fluorescence emission of the flake shown in (b) indicates the single-layer nature of the flake; Figure S2: Energy × Vertical Distance graph zoomed around the energy min-ima for WS${}_{2}$ Si${}_{3}$N${}_{4}$ interface; Figure S3: PL mapping (peak centred at 616.7 nm) of one region of the WS${}_{2}$ monolayer sample showing a continuous sample free of damage (left hole) and the high intensity of the signal on the suspended region in comparison with supported one; Figure S4: Optical image of region and Raman spectra of WS${}_{2}$ samples sus-pended and supported on the substrate, showing that there is no significant shifting of peak positions and the difference of the intensity between sus-pended and supported sample; Figure S5: Normalized Raman spectra of 1L-WS_2_ acquired in the suspended (upper panel) and supported (lower panel) regions. The vertical red dashed line highlights the slight blueshift for the suspended sample; Figure S6: Normalized Raman spectra of 1L-WS_2_ acquired in the suspended (upper panel) and supported (lower panel) regions. The vertical red dashed line highlights the slight redshift for the suspended sample; Figure S7: Normalized PL spectra of 1L-WS_2_ acquired in the suspended (blue trace) and supported (red trace) regions.
