# Supplementary material for: Substrate-Induced Changes on the Optical Properties of Single-Layer WS2
Source: Materials (Basel). 2023 Mar 24;16(7):2591. doi: 10.3390/ma16072591 (PMC10095963; doi:10.3390/ma16072591)
Supplement: Supplementary file 1 [file materials-16-02591-s001.zip › materials-2257159-supplementary.pdf]

Supporting Information for: Substrate Induced Changes on the Optical Properties of Single-Layer WS_2

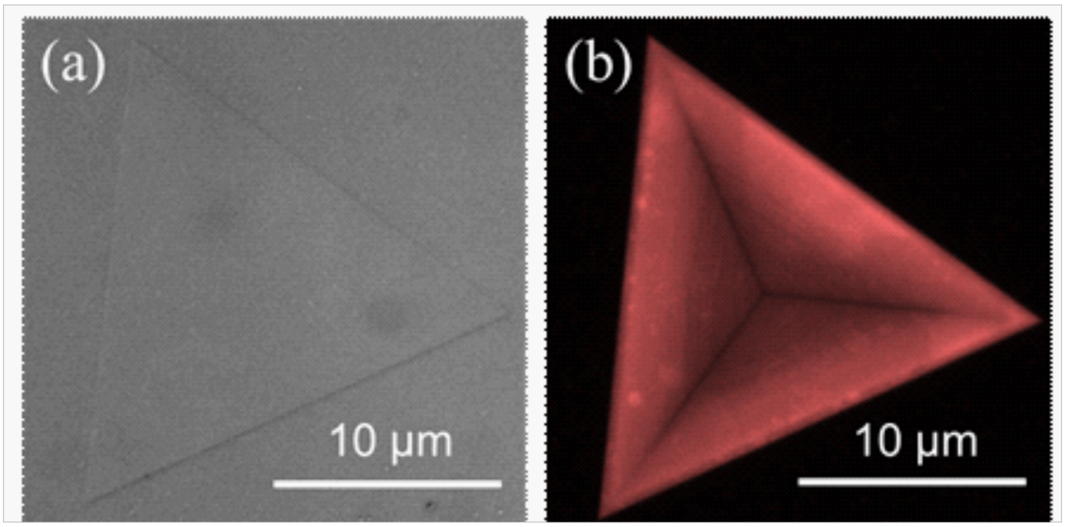

Figure S1: (a,b) Optical and fluorescence microscopy images of a typical CVD-grown WS_2 flake. The strong fluorescence emission of the flake shown in (b) indicates the single-layer nature of the flake.

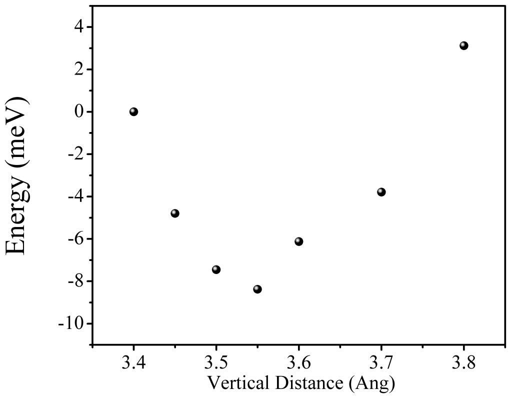

Figure S2: Energy × Vertical Distance graph zoomed around the energy min-ima for WS_2 Si_3N_4 interface.

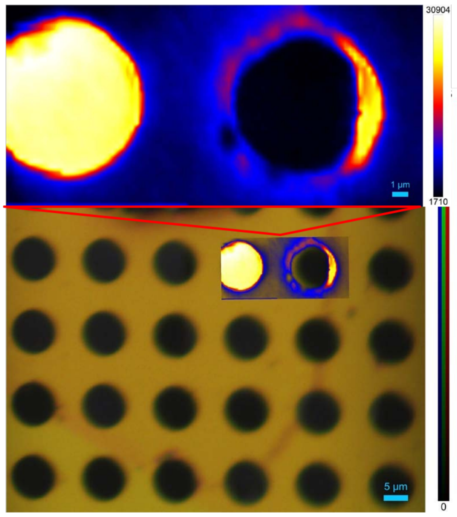

Figure S3: PL mapping (peak centred at 616.7 nm) of one region of the WS_2 monolayer sample showing a continuous sample free of damage (left hole) and the high intensity of the signal on the suspended region in comparison with supported one.

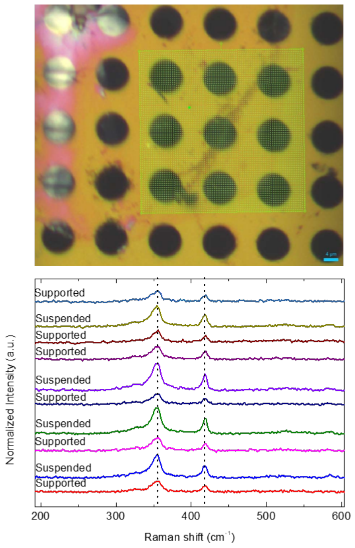

Figure S4: Optical image of region and Raman spectra of WS_2 samples sus-pended and supported on the substrate, showing that there is no significant shifting of peak positions and the difference of the intensity between sus-pended and supported sample.

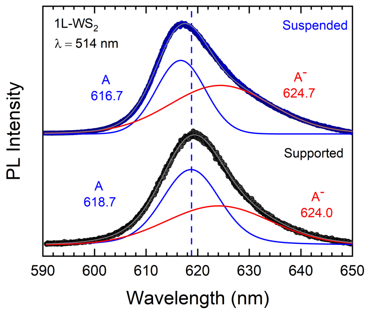

Figure S5: Normalized Raman spectra of 1L-WS2 acquired in the suspended (upper panel) and supported (lower panel) regions. The vertical red dashed line highlights the slight blueshift for the suspended sample.

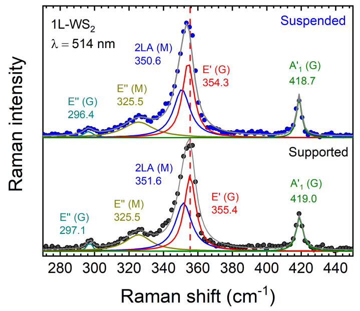

Figure S6: Normalized Raman spectra of 1L-WS2 acquired in the suspended (upper panel) and supported (lower panel) regions. The vertical red dashed line highlights the slight redshift for the suspended sample.

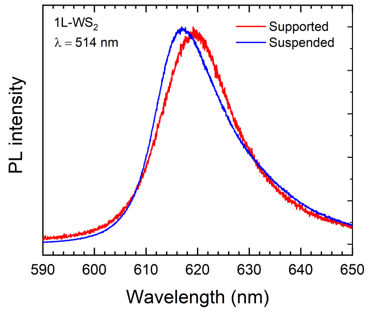

Figure S7: Normalized PL spectra of 1L-WS2 acquired in the suspended (blue trace) and supported (red trace) regions.
